# The Role of miRNAs in Parkinson’s Disease: A Systematic Review

**DOI:** 10.3390/ijms262412164

**Published:** 2025-12-18

**Authors:** Michalis Chrysanthou, Christiana C. Christodoulou, Eleni Zamba Papanicolaou

**Affiliations:** Neuroepidemiology Department, The Cyprus Institute of Neurology and Genetics, Nicosia 2371, Cyprus; michalisc@cing.ac.cy (M.C.); christianachr@cing.ac.cy (C.C.C.)

**Keywords:** Parkinson’s Disease, miRNA, biomarker, systematic review

## Abstract

Over the years, there has been extensive research conducted on Parkinson’s Disease (PD), a neurodegenerative disorder known for causing motor impairment and behavioral changes. In more recent years, the roles of dysregulated microRNAs (miRNAs) in PD pathology have been studied in the hopes of developing new diagnostic methods or even treatments. This systematic review pinpoints and examines studies between 2010 and 2024 that have identified significant dysregulation of miRNAs in patients with PD. Upon filtering out the search results by a series of exclusion criteria, this review was conducted using 56 relevant studies. These studies revealed a vast array of significantly dysregulated miRNAs identified in the samples of patients with PD, when compared to healthy controls. A number of these miRNAs, such as miR-29c-3p, are likely biomarkers for more accurate PD diagnosis, and many, such as miR-485-3p, were found to be involved in PD pathogenesis. With further research, miRNAs could become a helpful diagnostic and prognostic tool for PD, with some of them even being candidate therapeutic targets for future treatments.

## 1. Introduction

Parkinson’s Disease (PD) is a neurodegenerative disease that is caused by the accumulation of α-synuclein, leading to progressive loss of dopaminergic neurons in the substantia nigra region of the midbrain [1]. The disease is generally characterized by motor impairment, which becomes more severe over time. This consists of muscle rigidity that can make voluntary movements difficult and lead to involuntary tremors [2]. Additional symptoms related to PD include constipation, sleep disturbances, and an increased risk of mental problems, such as anxiety or cognitive impairment [2]. Despite extensive research on PD being conducted, the exact etiology of the disorder remains unknown [2]. This is likely due to PD having multifactorial origins in the majority of cases [3]. There are several known risk factors that can increase the chance of developing PD, which include, old age, exposure to toxic substances, head trauma, being male, and genetic susceptibility [1,2,3] and of these, the greatest risk factor is considered to be aging [2], with the prevalence of the disease expected to dramatically increase in the following decades [3]. PD can be divided into idiopathic PD (iPD) and familial PD (fPD). iPD constitutes approximately 95% of PD cases and is believed to be linked to several factors, while fPD has monogenic forms that can be inherited [4]. Genes associated with fPD include the Alpha-synuclein gene (*SNCA*), Vesicle Protein Sorting 35 gene (*VPS35*), PTEN Induced Kinase 1 gene (*PINK1*), Parkin gene (*PRKN*), Parkinsonism Associated Deglycase (*PARK7*), Phospholipase A2 Group VI gene (*PLA2G6*), Glucosylceramidase Beta gene (*GBA*), and Leucine–Rich Repeat Kinase 2 gene (*LRRK2*), among others [1,2]. Some of these genes, such as *SNCA* and *VPS35*, are inherited in an autosomal dominant pattern, while others, like *PINK1* and *PRKN*, are autosomal recessive [1,2]. Moreover, the genes *GBA* and *LRRK2* show variable penetrance [2].

MicroRNAs (miRNAs) are small non-coding RNAs consisting of around 21 to 24 nucleotides that bind to targeted mRNAs and inhibit their translation into proteins. Some miRNAs are expressed only in specific tissues, while others can be found in all parts of the body [5,6]. The expression of miRNAs has been noted to be dysregulated in several neurodegenerative diseases, including PD, with many even playing a role in the development and progression of the disorder [5]. It should be noted that miRNA dysregulation could be caused by other, independent mechanisms. A known potential cause for this is mutations in genes that encode for certain proteins, which are involved in miRNA biogenesis. Such mutations can result in dysregulation of pre-miRNA splicing and maturation, leading to altered miRNA expression. Dysregulation in the expression of genes that are essential in miRNA biogenesis has even been linked to the development or pathological mechanism of serious conditions such as cancer and infertility [7,8].

While this study focuses on miRNAs mainly related to PD and perhaps a few other neurodegenerative diseases, there are known miRNAs that play central roles in the nervous system, which are involved in all types of neurodegeneration. These include miRNAs responsible for the homeostasis of the central nervous system (CNS) (miR-124, miR-125, and miR-132) and miRNAs related to immunity (miR-21, miR-146a, and miR-155) [9].

Due to their role in regulating gene expression, as well as their involvement in pathogenesis, some miRNAs have also been considered as potential therapeutic targets for certain neurodegenerative diseases, with examples including miR-455-3p and miR-125b for Alzheimer’s Disease (AD) and miR-23a for Amyotrophic Lateral Sclerosis (ALS), as well as miR-155a and miR-146a for Multiple Sclerosis (MS) [10]. This usually consists of trying to mediate the dysregulation of key miRNAs, either by using miRNA sponges to reduce upregulated miRNAs or with miRNA mimics to increase downregulated miRNAs, accordingly [10]. There are several miRNA-based treatments currently being tested on animal and cell models with promising results for multiple neurodegenerative diseases. There are, however, some limitations to overcome, such as achieving efficient transportation of mimic miRNAs to the targeted tissues and passing through the blood–brain barrier (BBB) [10]. Certain miRNAs that regulate genes linked to PD, such as *SNCA*, *LRRK2*, and *PARK2*, have even been considered to play a role in the causation of PD [11]. Moreover, differences in miRNA expression have been shown to predict PD development, as well as the development of isolated rapid eye movement sleep behavior disorder (iRBD) [12].

In order to better understand the diagnostic value and therapeutic potential of miRNAs in PD, a review of the available literature needs to be conducted. This systematic review focuses on PD patient-derived studies involving miRNAs as potential biomarkers and therapeutic targets. The aim of this review is to summarize the available literature and evaluate which miRNAs are more closely related to PD.

## 2. Materials and Methods

### 2.1. Studies Included

**Figure 1:**
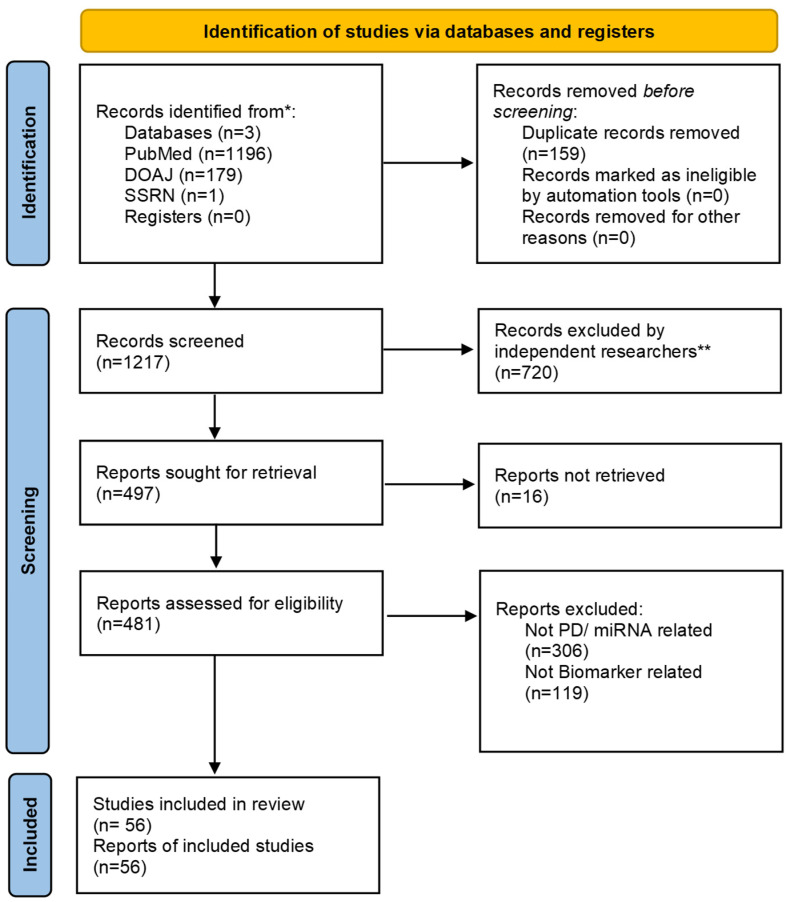
The identification and screening of studies included in this review, displayed using the PRISMA 2020 flow diagram for systematic reviews. * If feasible, report the number of records identified from each database or register searched. ** If automation tools were used, indicate how many records were excluded by a human and how many were excluded by automation tools.

To better understand the involvement of miRNAs in PD development and progression, a review of the literature was conducted, utilizing electronic databases such as PubMed, Directory of Open Access Journals (DOAJ), and Social Science Research Network (SSRN). The search spanned studies between 2010 and 2024. The following prompt words were used ‘’Parkinson’s Disease and miRNA’’. The research papers identified were then screened through their abstract to see if they fit the purposes of the study, with articles found to be eligible studied more thoroughly. Studies that were unrelated to miRNAs or focused solely on other neurodegenerative diseases were excluded. The review consisted of full-text articles written in the English language. The initial search yielded 1376 research articles. Due to this high number of results, the decision was made to focus on studies conducted on human patients with PD, with the main goal of investigating potential miRNA biomarkers for PD. Studies that involved animal models or cell models were not included. Additionally, experimental studies that solely analyzed the function of miRNAs were excluded. After filtering out studies that did not fulfil the inclusion criteria, a total of 56 articles were used in this review. The selection process can be seen in Figure 1. This systematic review was based on the PRISMA guidelines [13] (https://www.prisma-statement.org/prisma-2020 accessed on 12 December 2025), and the PRISMA checklist can be found in Supplementary Data in Table S1.

### 2.2. Assessment of Risk Bias in Included Studies

To better assess the validity of our review and detect potential limitations, the risk bias of the studies included was estimated through the use of quality assessment tools. Both case–control studies and cohort studies included were examined using the NIH study quality assessment tools https://www.nhlbi.nih.gov/health-topics/study-quality-assessment-tools (accessed on 31 October 2025).

## 3. Results

### 3.1. Studies Included Within the Review

After the search and selection process, this review was mainly conducted using case-control studies [14,15,16,17,18,19,20,21,22,23,24,25,26,27,28,29,30,31,32,33,34,35,36,37,38,39,40,41,42,43,44,45,46,47,48,49,50,51,52,53,54,55,56,57,58,59,60,61,62,63,64,65,66], as well as some cohort studies [19,27,43,45,46,54,56,58,67,68,69]. To maintain the relevance of the research to the subject, a series of exclusion criteria was applied. These included (i) systematic reviews or other meta-analyses, (ii) studies on other neurodegenerative diseases, (iii) studies unavailable in English, (iv) studies based on bioinformatics data, (v) studies unrelated to miRNA or PD, (vi) retracted articles, and (vii) studies with no human participants. It should be noted that studies that were conducted on human participants but also used animal or cell models to verify their results were not excluded.

### 3.2. Characteristics of PD Patient Studies

A total of 56 research articles were studied for the purposes of this review. The vast majority of these studies involved the identification of miRNA biomarkers that can improve prognosis or diagnosis by differentiating between PD and healthy controls, or other neurological conditions. A notable number of these miRNAs were detected in a variety of tissues derived from PD patients, including whole blood samples, serum, plasma, cerebrospinal fluid (CSF), peripheral blood mononuclear cells (PBMCs), induced pluripotent stem cells (iPSCs), saliva, leukocytes, and post-mortem brain tissues. In some studies, the role of the identified miRNAs in PD pathogenesis was also researched. This results in some miRNA biomarkers being labelled as potential therapeutic targets for future research. All studies included were performed on human samples; however, some studies also used animal cell models to verify their results. The full list of the studies used in the review, along with their characteristics and findings, is illustrated in Table 1.

### 3.3. Findings of Included Studies

The main finding across the reviewed studies was the identification of a variety of dysregulated miRNAs from different tissues that can be utilized as biomarkers to distinguish PD from healthy controls or other neurodegenerative disorders. Examples of effective biomarkers that were found to reliably distinguish PD from other disorders and healthy controls include the miRNAs miR-29a-3p, miR-29c-3p, and miR-6756-5p, which were detected in the saliva, making them a non-invasive diagnostic tool (Jiang et al., 2021) [49]. The most significant of these was miR-29a-3p. Biomarkers can also be used to monitor disease progression, such as miR-376a, whose higher concentration has been associated with more severe PD pathology (Baghi et al., 2020) [31]. In many cases, miRNAs were found to play a role in PD pathogenesis through the pathways they regulate. Due to this, some of these studies suggest certain miRNAs as candidates for the development of treatments or for monitoring the progress of already existing treatments. This can be seen in the case of miR-155-5p, which showed milder dysregulation when a higher dosage of Levodopa was administered (Caggiu et al., 2018) [53]. The functional significance, biological function of the identified miRNAs, along with their validation status, can be seen in Supplementary Table S5.

## 4. Discussion

In recent years, the involvement of miRNA in the pathogenesis of neurodegenerative disorders has been extensively researched. Through these studies, the connection between miRNA dysregulation and the development of neurodegenerative disease, including PD, has been established [10,12].

In this review, we have summarized the results of several such studies focused on PD, in order to compile our knowledge of miRNA biomarkers that can improve our diagnostic and prognostic capabilities for this disorder. Several miRNA biomarkers were identified, with many of them playing an active role in PD pathology. The involvement and significance of these miRNAs varied, with some being identified and studied more consistently than others. A number of these miRNAs have also been suggested as potential therapeutic targets.

### 4.1. miRNA Biomarkers in PD

For example, two individual studies by Lin et al. [15] and Nair & Ge [40] investigated miR-485-3p as a promising biomarker that could distinguish patients with PD from healthy controls, as well as patients with AD [15,40]. In both of these studies, miR-485-3p expression was found to be significantly upregulated in post-mortem brain tissue samples and serum obtained from patients with PD. This miRNA was also found to regulate inflammation and to be involved in PD progression [15]. These results are in accordance with the literature, as the dysregulation of miR-485-3p, along with its direct involvement in AD and PD development through the inflammatory processes it regulates, has been researched in other studies [95,96]. Moreover, inhibition of miR-485-3p has been shown to reduce inflammation and amyloid plaque aggregation in AD mice, while helping preserve cognitive function [95]. The known validated target genes for miR-485-3p so far are AKT serine/threonine kinase 3 (*AKT3)* and Peroxisome proliferator-activated-receptor-γ cofactor 1-alpha (*PGC1α*), which are involved in neuroinflammation and apoptosis [96].

Another prominent miRNA that was mentioned across multiple studies [49,65,67] was miR-29c-3p. This miRNA was found to be significantly dysregulated in the plasma, CSF, saliva, and leukocytes of patients with PD. The differential expression of miR-29c-3p was found to distinguish between PD and other disorders, namely multiple system atrophy (MSA) and essential tremor (ET) [49]. Moreover, miR-29c-3p was shown to distinguish between sPD- and LRRK2-caused PD [67]. These results, along with its presence in saliva, make miR-29c-3p a useful and easily accessible, non-invasive biomarker for PD diagnosis. There are multiple known gene targets for miR-29c-3p, which include Mesoderm-specific transcript (*MEST*) and Secreted protein acidic and rich in cysteine (*SPARC*), which are known to be involved with colorectal and gastric cancer [97,98]. MiR-29c-3p was also shown to target the Beta-site Amyloid precursor protein Cleaving Enzyme 1 (*BACE1*) in mice and is, thus, believed to play a role in AD progression [99].

A study conducted by Ravanidis et al. [20] also found that miRNAs could distinguish between different types of PD, including sPD, GBA-PD, and A53T-PD (*SNCA* mutation). Furthermore, this research found a pair of miRNAs (miR-136-3p and miR-433-3p) that were dysregulated in all types of PD studied. miR-136-3p seems to be more prominent as it was mentioned in two additional studies reviewed [27,37]. These studies also identified the diagnostic potential of miR-136-3p in regards to patients with PD and healthy controls; however, it was also found to distinguish between PD and AD [27]. This miRNA biomarker was detectable in samples derived from CSF and blood plasma. Known gene targets for miR-136-3p include Kruppel-like factor 7 (*KLF7*), whose downregulation suppresses glial tumor formation [100], and Phosphatase and TENsin homolog (*PTEN*), which regulates bone and blood vessel formation [101].

### 4.2. Potential Treatments and miRNA Therapeutic Targets

The involvement of miRNAs in PD progression and their therapeutic potential was highlighted in some of the studies reviewed. Notably, a pair of studies [24,69] included showed that exercise was an effective treatment for improving cognitive and motor function in patients with PD, with the expression of miRNAs being altered in the process [24,69]. This hints at certain miRNAs being not only biomarkers, but also likely targets for future therapy, although further research is required to determine the efficiency of such treatments.

Treatment with Levodopa was also shown to alter miRNA expression in patients with PD [45,53]. More specifically, the expression of three miRNAs, namely miR-29a-3p, miR-30b-5p, and miR-103a-3p, was found to be significantly increased in patients with PD treated with Levodopa, in comparison to untreated patients and controls [45]. Moreover, higher Levodopa dosage was observed to better medicate the dysregulation of miR-146a-5p and miR-155-5p in the serum of patients with PD [53]. These results suggest the involvement of the two miRNAs in disease progression and indicate their potential as biomarkers or even therapeutic targets. From the literature, we know that miR-146a-5p has anti-inflammatory properties, as it regulates M1 macrophages by targeting CD80 [102]. Meanwhile, miR-155-5p is known to prompt inflammation by upregulating Interleukin-8 (IL-8) [103], which could explain why it is upregulated in PD, unlike miR-146a-5p, which is reduced [53].

### 4.3. Strengths, Limitations, and Future Work

The main strength of this systematic review is the wide array of different miRNAs researched throughout the studies included. This high number of miRNAs resulted in an abundance of candidate biomarkers for the diagnosis and prognosis of PD. Furthermore, these miRNAs were acquired from a variety of different samples, such as blood, CSF, and saliva, making these biomarkers versatile and, in some cases, non-invasive. Another advantage of this review would be the use of a variety of methods to identify miRNAs and their involvement in PD pathogenesis. The most notable of these included qRT-PCR, miRNA TaqMan assays, miRNA target prediction, and statistical analysis of the results to determine the significance of dysregulated miRNAs.

Some limitations were encountered in the conduction of the review. While most studies included had a large number of patients with PD and controls recruited or even multiple cohorts, some studies were conducted using notably low cohorts, which could make their results less reliable. In addition, in a few studies, some information regarding the subject’s age or gender was either unavailable or unclear. Furthermore, while some miRNAs were able to distinguish PD from healthy controls, it was not always tested whether this was exclusive to PD or a common dysregulated miRNA between multiple neurodegenerative diseases. Another potential limitation from the included studies comes from the fact that many of the results gathered have not been validated with functional evidence or by being replicated in other cohorts, which would have made these findings more reliable. The most notable limitation of the review, however, was the lack of follow-up seen in the majority of the studies included. While there was an abundance of pilot studies that identified potential miRNA biomarkers and therapeutic targets, very few of these had follow-up studies to confirm these results. The exclusion of experimental studies from our review also plays a role in this, as such studies could have provided us with a better overview of the mechanisms and pathways through which certain miRNAs are involved with PD.

Future work should focus on validating the diagnostic, prognostic, and therapeutic value of the identified miRNAs that were associated with PD in either larger PD cohorts and in other neurodegenerative disorders to determine the specificity and sensitivity of this miRNA to estimate the potential of miRNAs identified as possible therapeutic targets, research on human cell models can be undertaken, with any promising results potentially moving on to clinical trials. Other studies to explore the involvement of more miRNAs in PD could also be conducted, since there are several more miRNAs that are yet to be researched. Furthermore, in order to gain a more complete image of the role of miRNAs, a follow-up review could be conducted, focusing on the omitted experimental studies to complement the findings of this study.

## 5. Conclusions

Based on the results gathered from a multitude of studies reviewed, we can conclude that miRNAs are deeply involved in both the progression and mediation of PD. There is an abundance of evidence pointing to miRNA dysregulation being a viable diagnostic biomarker for accurately diagnosing PD, even among other degenerative neuropathies, and for monitoring disease progression. Additionally, a number of miRNAs have the potential to be utilized for the treatment of patients with PD, although further research on this matter is required.

## Figures and Tables

**Table 1:** Summarization of the studies involving the diagnostic and therapeutic potential of miRNAs in PD.

First Author, Year, and Country	Study Type, Subjects, and Ethnicity	Mean Age at Sample Collection (Years)	Sample Type	Methods	Clinical Outcome, Analysis, and Effect Estimation	*p*-Value	Results Validation
Salemi et al., 2022 (Italy) [14]	Case–control PD patients: (*n* = 16) Males/Females: (*n* = 10/6) Controls: (*n* = 14) Males/Females: (*n* = 10/4) Ethnicity: Italian (Sicily)	PD: 68.00 ± 6.47 Controls: 71.94 ± 13.19	PBMCs	miRNA extraction, miRNA sequencing, and data analysis	Upregulated in PD: miR-1275, miR-23a-5p, miR-432-5p, miR-4433b-3p, miR-4443. Downregulated in PD: miR-142-5p, miR-143-3p, miR-374a-3p, miR-542-3p, miR-99a-5p.	NR	Yes (enrichment analysis)
Fazeli et al., 2020 (Iran) [25]	Case–control PD patients: (*n* = 30) Males/Female: (*n* = 21/9) Controls: (*n* = 14) Males/Females: (*n* = 11/3) Ethnicity: N/A	PD: 62 ± 11.11 Controls: 63.93 ± 11.96	PBMCs	PBMC isolation RNA extraction qRT-PCR Statistical analysis	miR-27a-3p expression was decreased in patients with PD, with its concentration being lower according to the disease progression. SRRM2 expression was also reduced in a similar way.	0.015 *	Yes (enrichment analysis)
Baghi et al., 2020 (Iran) [31]	Case–control PD patients: (*n* = 33) Males/Females: (*n* = 23/10) Controls: (*n* = 25) Males/Females: (*n* = 16/9) Ethnicity: N/A	PD: 62.904 ± 11.430 Controls: 60.28 ± 10.125	PBMCs	PBMC isolation Candidate miRNA selection Cell culture neurotoxin treatment Cell viability assessment Flow Cytometry Intracellular ROS Measurement RNA extraction, synthesis of cDNA RT-PCR Statistical analysis	miR-376a expression increased in patients with PD, with higher concentrations being associated with greater disease severity.	<0.001 *	Yes (cell model)
Behbahanipour et al., 2019 (Iran) [34]	Case–control PD patients: (*n* = 36) Males/Females: (*n* = 25/11) Controls: (*n* = 16) Males/Females: (*n* = 11/5) Ethnicity: N/A	PD: 61.3 ± 11.4 Controls: 62.5 ± 12.4	PBMCs	PBMC isolation RNA extraction, and quality control qRT-PCR Statistical analysis miRNA target analysis Functional enrichment and pathway analysis	miR-885-5p, miR-361-5p, and miR-17-5p were significantly dysregulated in the blood of patients with PD. miR-361-5p and miR-17-5p can distinguish between early and late-stage patients with PD.	<0.001 * 0.036 * 0.034 * <0.001 * 0.116 0.009 * 0.332 <0.001 * 0.416 0.023 * 0.021 *	Yes (pathway and enrichment analysis)
Serafin et al., 2015 (Italy) [45]	Case–control/cohort L-dopa PD patients: (*n* = 36) Drug-naïve PD patients: (*n* = 10) Controls: (*n* = 46) Ethnicity: N/A	NR	PBMCs	RNA isolation qRT-PCR Relative Quantification of miRNAs Statistical analysis Prioritization of miRNA targets	miR-29a-3p, miR-30b-5p, and miR-103a-3p were significantly overexpressed in patients treated with L-dopa.	0.005 * 0.002 * <0.0001 *	No
Caggiu et al., 2018 (Italy) [53]	Case–control PD patients: (*n* = 37) Males/Females: (*n* = 20/17) Controls: (*n* = 43) Males/Females: (*n* = 16/27) Ethnicity: Italian (Sardinian)	PD: 71.3 ± 9.6 Controls: 60 ± 13.14	PBMCs	miRNAs cDNA Synthesis qPCR Heat Maps Statistical analysis	miR-155-5p was upregulated in PD samples vs. controls, while miR-146a-5p expression was significantly reduced. Patients receiving a higher Levodopa dose showed a milder increase in miR-155-5p expression.	<0.0001 * 0.0015 *	No
Baghi et al., 2021 (Iran) [59]	Case–control PD patients: (*n* = 20) Males/Females: (*n* = 12/8) Controls: (*n* = 20) Males/Females: (*n* = 14/6) Ethnicity: N/A	PD: 61.7 ± 12.55 Controls: 58.45 ± 9.39	PBMCs	Pathway enrichment analysis Cell culture Cell transfection MPP+ treatment Intracellular ROS Measurement Annexin V Staining RNA extraction qRT-PCR Statistical analysis	The levels of miR-193b were significantly increased in PD. miR-193b was found to be involved in PD progression through the PGC1a-FNDC5-BDNF pathway.	<0.0001 *	Yes (cell model)
Lin et al., 2022 (China) [15]	Case–control PD patients: (*n* = 92) AD patients: (*n* = 66) Controls: (*n* = 64) Ethnicity: N/A	NR	Serum	RNA extraction qRT-PCR Statistical analysis Diagnostic performance of serum miR-485-3p in patients with PD	Significant upregulation of miR-485-3p in patients with PD serum compared to AD and control samples.	<0.001 * <0.001 *	Yes (animal models)
Citterio et al., 2023 (Italy) [16]	Case–control PD patients: (*n* = 45) Males/Females: (*n* = 26/19) Controls: (*n* = 49) Males/Females: (*n* = 25/24) Ethnicity: N/A	PD: 67.30 ± 9.02 Controls: 65.49 ± 12.15	Serum	RNA extraction qRT-PCR Statistical analysis	Increased levels of miR-7-1-5p and miR-223-3p compared to healthy controls.	0.0004 * 0.0006 *	No
He et al., 2021 (China) [68]	Cohort PD stage II: (*n* = 8) Males/Females: (*n* = 5/3) PD stage III: (*n* = 42) Males/Females: (*n* = 26/16) PD stage IV: (*n* = 22) Males/Females: (*n* = 12/10) Controls: (*n* = 31) Males/Females: (*n* = 17/14) Ethnicity: N/A	PD II: 59.75 ± 7.55 PD III: 61.62 ± 7.6 PD IV: 64.73 ± 8.14 Controls: 63.94 ± 7.45	Serum	EV extraction and validation RNA isolation qRT-PCR RNA sequencing and data pre-processing Differential expression analysis and WGCNA Statistical analysis	PD serum derived EVs, revealed dysregulated miRNAs: miR-374a-5p, miR-374b-5p, miR-199a-3p, miR-28-5p, miR-22-5p, miR-151a-5p. miRNAs expression fluctuated between PD stages.	< 0.0001 * < 0.0001 * < 0.0001 * < 0.0001 * < 0.0001 * < 0.0001 *	No
Da Silva et al., 2021 (Brazil) [24]	Case–control PD patients: (*n* = 4) Males/Females: (*n* = 4/0) Controls: (*n* = 4) Males/Females: (*n* = 4/0) Ethnicity: N/A	PD: 66.25 ± 12.97 Controls: 63.50 ± 9.60	Serum	RNA extraction qRT-PCR Prediction of target genes Interval Training Program Statistical analysis	Expression of miR-106a-5p, miR-103a-3p, and miR-29a-3p increased in patients with PD patients and controls after exercise. Increased concentrations of these miRNAs were correlated to better cognitive function.	0.04 *	No
Li et al., 2020 (China) [28]	Case–control PD patients: (*n* = 80) Males/Females: (*n* = 42/38) Controls: (*n* = 60) Males/Females: (*n* = 31) Ethnicity: N/A	PD: 64.6 ± 7.54 Control: 64.0 ± 7.29	Serum	Cell culture and treatment RNA extraction qRT-PCR ELISA Luciferase Activity Assay Statistical analysis	miR-150 was significantly downregulated patients with PD.	<0.001 *	Yes (cell model)
Ma et al., 2016 (China) [33]	Case–control PD patients: (*n* = 138) Males/Females: (*n* = 75/63) Controls: (*n* = 112) Males/Females: (*n* = 61/51) Ethnicity: N/A	PD: 29.36 ± 13.25 Controls: 31.23 ± 19.16	Serum	RNA isolation qRT-PCR Statistical analysis	Significantly dysregulated in patients with PD: miR-146a-5p, miR-214, miR-221, miR-29c.	0.0042 * <0.001 * <0.001 * 0.0037 *	No
Bai et al., 2017 (China) [35]	Case–control PD patients: (*n* = 80) Males/Females: (*n* = 48/32) AD patients: (*n* = 30) Males/Females: (*n* = 14/16) PD controls: (*n* = 80) Males/Females: (*n* = 48/32) AD controls: (*n* = 30) Males/Females: (*n* = 18/12) Ethnicity: N/A	PD: 64.0 ± 5.8 PD controls: 63.3 ± 5.4 AD: 78.6 ± 9.5 AD controls: 42.6 ± 11.9	Serum	RNA extraction qRT-PCR Statistical analysis	miR-29s expression was greatly decreased in the serum of patients with PD.	< 0.01 *	No
Jin et al., 2018 (China) [36]	Case–control PD patients: (*n* = 46) AD patients: (*n* = 40) MSA patients: (*n* = 35) Controls: (*n* = 46) Ethnicity: N/A	NR	Serum	qRT-PCR Cell culture Plasmid Construction miRNA mimics and inhibitors Luciferase Assay Western Blot Statistical analysis	miR-520d-5p was overexpressed in the serum of patients with PD compared to controls but not significantly when compared to MSA and AD.	0.0011 *	Yes (cell model)
Dong et al., 2016 (China) [38]	Case–control PD patients: (*n* = 122) Males/Females: (*n* = 62/60) Controls: (*n* = 104) Males/Females: (*n* = 51/53) Ethnicity: N/A	PD: 67.6 (7.5) Controls: 66.0 (5.3)	Serum	RNA isolation Solexa sequencing In silico analysis qRT-PCR Statistical analysis	Thirty miRNAs found to be differentially expressed in serum PD, the four most significant were: miR-141, miR-214, miR-146b-5p, miR-193a-3p.	<0.0001 *	No
Zhang et al., 2024 (China) [69]	Cohort PD patients exercise: (*n* = 13) Males/Females: (*n* = 6/7) PD patient controls: (*n* = 6) Males/Females: (*n* = 4/2) Ethnicity: N/A	PD exercise: 53.231 (6.735) PD controls: 52.667 (7.685)	Serum	Exercise intervention MiRNAs extraction Small RNA sequencing qRT-PCR Gene ontology and KEGG enrichment analysis Statistical analysis	Between the patients with PD who exercise and those who did not, ten miRNAs were found to be significantly upregulated: miR-1268a, miR-181a-2-3p, miR-320c, miR-320d, miR-619-5p, miR-877-5p, miR-115-5p, miR-116-5p, miR-209-3p, miR-255-5p. While another was downregulated: miR-181-3p.	2.19 × 10^−5^ * 0.0002 * 3.26 × 10^−5^ * 5.91 × 10^−5^ * 9.52 × 10^−6^ * 0.0004 * 3.01 × 10^−5^ * 3.51 × 10^−7^ * 0.0003 * 0.0005 * 0.0002 *	No
Vallelunga et al., 2021 (Italy) [50]	Case–control PD patients: (*n* = 51) MSA patients: (*n* = 52) Controls: (*n* = 56) Ethnicity: N/A	NR	Serum	miRNAs quantification Data analysis Statistical analysis Target prediction	miR-96-5p concentration was significantly increased in PD and MSA samples vs. controls. miR-339-5p distinguished between MSA and PD, but only reliable in female patients.	<0.0001 * <0.01 *	Yes (validation of previous study by the same researchers [51])
Li et al., 2021 (China) [54]	Case–control/cohort pPD patients: (*n* = 25) Males/Females: (*n* = 13/12) dnPD patients: (*n* = 20) Males/Females: (*n* = 9/11) aPD patients: (*n* = 24) Males/Females: (*n* = 12/12) Controls: (*n* = 21) Males/Females: (*n* = 10/11) Ethnicity: N/A	pPD: 68.00 (63.00–70.00) dnPD: 65.00 (64.00–68.00) aPD: 66.50 (63.25–69.00) Controls: 64.00 (62.00–66.00)	Serum	qRT-PCR Clinical assessment Statistical analysis	miR-31 was significantly increased in aPD vs. controls and dnPD patient serum. miR-214 was increased in the pPD group compared to controls and aPD.	0.005 * 0.001 * 0.003 *	No
Vallelunga et al., 2014 (Italy) [51]	Case–control PD patients: (*n* = 25) Males/Females: (*n* = 13/12) MSA patients: (*n* = 25) Males/Females: (*n* = 12/13) Controls: (*n* = 25) Males/Females: (*n* = 13/12) Ethnicity: N/A	NR	Serum	RNA isolation qRT-PCR TaqMan Low Density Array Data analysis miRNA target prediction Gene ontology analysis	PD samples: Three miRNAs were upregulated and three were downregulated compared to controls. Upregulated: miR-223, miR-324-3p, miR-24. Downregulated: miR-339-5p, miR-30c, miR-148b. MSA samples: Four miRNAs upregulated and one was downregulated when compared to controls. Upregulated: miR-223, miR-324-3p, miR-24, miR-148b. Downregulated: miR-339-5p. Three miRNAs that distinguish between PD and MSA: miR-24, miR-34b, miR-148b.	0.03 * 0.036 * 0.039 * 0.006 * 0.036 * 0.00008 * 0.00009 * 0.0003 * 0.0002 * 0.032 * 0.00004 * 0.012 * 0.0006 *	Yes (partially validated by other study from the same researchers [50])
Soto et al., 2023 (Spain) [58]	Case–control/cohort Cohort 1: (*n* = 99) iPD patients: (*n* = 19) Males/Females: (*n* = 12/7) L2NMC-: (*n* = 20) Males/Females: (*n* = 8/12) L2NMC+: (*n* = 20) Males/Females: (*n* = 12/8) L2PD: (*n* = 20) Males/Females: (*n* = 12/8) Controls: (*n* = 40) Males/Females: (*n* = 28/12) Cohort 2: (*n* = 39) L2PD: (*n* = 19) Males/Females: (*n* = 8/11) Controls: (*n* = 20) Males/Females: (*n* = 8/12) Ethnicity: N/A	iPD 1: 63.53 ± 11.77 L2NMC- 1: 52.30 ± 10.12 L2NMC+ 1: 60.50 ± 14.49 L2PD 1: 65 ± 10.90 Controls 1: 65.48 ± 11.69 L2PD 2: 64.47 ± 11.34 Controls 2: 63.65 ± 10.75	Serum	RNA isolation Genome-wide miRNA analysis qRT-PCR ROC analysis Biological enrichment analysis	Seven miRNAs were found to be dysregulated in L2NMC mutation carriers, with miR-8069 being novel. miR-4505 was identified in the blood of patients with L2PD, while miR-185-5p and miR-221-3p could discriminate between PD and controls.	<0.05 *	Yes (enrichment analysis)
Shu et al., 2020 (China) [63]	Case–control PD patients: (*n* = 82) Males/Females: (*n* = 52/30) Controls: (*n* = 44) Males/Females: (*n* = 27/17) Ethnicity: N/A	PD: 68.53 ± 7.53 Controls: 66.24 ± 8.62	Serum	qRT-PCR Statistical analysis	Serum PD showed a notable decrease in miR-132-3p and miR-146a-5p expression, more evident in severe cases of PD.	<0.01 * <0.01 *	No
Chen et al., 2021 (China) [17]	Case–control Cohort 1: (*n* = 156) PD patients: (*n* = 78) Males/Females: (*n* = 42/36) Controls: (*n* = 78) Males/Females: (*n* = 40/38) Cohort 2: (*n* = 42) PD patients: (*n* = 27) Males/Females: (*n* = 13/14) Controls: (*n* = 15) Males/Females: (*n* = 7/8) Cohort 3: (*n* = 112) PD patients: (*n* = 46) Males/Females: (*n* = 13/33) MSA: (*n* = 21) Males/Females: (*n* = 7/14) Controls: (*n* = 45) Males/Female: (*n* = 18/27) Ethnicity: N/A	C1 PD: 60.80 (58.64–62.96) C1 controls: 59.68 (58.22–60.14) C2 PD: 60.11 (56.51–63.71) C2 controls 59.92 (55.32–64.52) C3 PD: 63.09 (60.19–65.99) C3 MSA: 61.86 (58.75–64.97) C3 controls: 61.54 (59.96–63.12)	Plasma	RNA extraction Polyadenylation qRT-PCR Statistical analysis	Thirty-two miRNAs were dysregulated in plasma samples. Seven selected as biomarker candidates: miR-432-5p, miR-133b, miR-320a, miR-4454, miR-221-3p, miR-627-5p, miR-205.	0.028 * 0.041 * 0.024 * 0.028 * 0.05 * 0.016 * 0.032 *	Yes (validation cohort)
Xie et al., 2022 (China) [18]	Case–control PD patients: (*n* = 30) Males/Females: (*n* = 17/13) Controls: (*n* = 30) Males/Females: (*n* = 17/13) Ethnicity: N/A	PD: 59.97 ± 7.89 Controls: 58.20 ± 9.36	Plasma	EVs isolation and TEM DLS measurements Cell culture Changes of SH-SY5Y cells after MPP+ induction Western Blot Data analysis	Plasma EV concentrations of these miRNAs were altered: miR-15b-5p, miR-30c-2-3p, miR-138-5p, miR-338-3p, miR-106b-3p, miR-431-5p, miR-146a-5p, miR-411-5p.	0.0065 * 0.0035 * 0.0106 * 0.0224 * 0.0169 * 0.0075 * 0.4991 0.1444	Yes (cell model)
Ravanidis, Bougea, Papagiannakis, Maniati, et al., 2020 (Greece) [20]	Case–control/cohort iPD patients: (*n* = 99) Males/Females: (*n* = 55/44) GBA-PD patients: (*n* = 27) Males/Females: (*n* = 14/13) A53T-PD patients: (*n* = 26) Males/Females: (*n* = 11/15) Controls: (*n* = 101) Males/Females: (*n* = 23/78) Ethnicity: N/A	iPD: 67.13 ± 12.42 GBA-PD: 60.00 ± 10.87 A53T-PD: 51.83 ± 11.59 Controls: 61.57 ± 10.55	Plasma	miRNA isolation from plasma and qRT-PCR analysis List of brain-enriched miRNAs Statistical analysis	Each PD type had its own profile of dysregulated miRNAs. Common miRNAs between all types were: miR-136-3p, miR-433-3p.	0.000003 * 0.005 *	Yes (validated by other study from the same researchers [19])
Grossi et al., 2021 (Italy) [23]	Case–control PD patients: (*n* = 15) Males/Females: (*n* = 15/0) Controls: (*n* = 14) Males/Females: (*n* = 14/0) Ethnicity: N/A	PD: 75.7 ± 3.0 Controls: 78.5 ± 7.3	Plasma	Plasma pre-analytical processing EV preparations from plasma Western Blot AFM Imaging and Size distribution EV subpopulations Purity assessment Total RNA isolation Statistical analysis	miR-34a-5p expression in PD plasma was significantly upregulated compared to controls.	<0.05 *	No
Hsu et al., 2024 (Taiwan) [27]	Case–control/cohort Cohort 1: (*n* = 123) PD patients: (*n* = 37) PD-MCI: (*n* = 23) PDD: (*n* = 23) Controls: (*n* = 40) Cohort 2: (*n* = 120) PD patients: (*n* = 30) PD-MCI: (*n* = 30) PDD: (*n* = 30) Controls: (*n* = 30) Ethnicity: N/A	PD 1: 64.78 ± 12.51 PD-MCI 1: 67.70 ± 7.15 PDD 1: 72.00 ± 5.52 Controls 1: 69.08 ± 6.05 PD 2: 69.67 ± 7.03 PD-MCI 2: 70.13 ± 6.75 PDD 2: 75.20 ± 6.92 Controls 2: 66.67 ± 5.14	Plasma	Cognitive assessments Plasma collection RNA extraction Plasma miRNA sequencing BOLD selector data analysis Statistical analysis	Significantly upregulated in PD vs. controls: miR-22-3p, miR-124-3p, miR-136-3p, miR-154-5p, miR-323a-3p. miRNAs distinguished between non-demented patients with PD and patients with PD with MCI: miR-203a-3p, miR-626, miR-662, miR-3182, miR-4274, miR-4295.	NR	Yes (validation cohort)
Yang et al., 2019 (China) [29]	Case–control Cohort 1: (*n* = 667) PD patients: (*n* = 269) Males/Females: (*n* = 157/112) ND controls: (*n* = 176) Males/Females: (*n* = 105/71) Healthy controls: (*n* = 222) Males/Females: (*n* = 130/92) Cohort 2: (*n* = 345) PD patients: (*n* = 142) Males/Females: (*n* = 79/63) ND controls: (*n* = 105) Males/Females: (*n* = 56/49) Healthy controls: (*n* = 98) Males/Females: (*n* = 54/44) Ethnicity: Chinese	PD1: 66.10 ± 0.61 NDC1: 66.15 ± 0.74 HC1: 66.16 ± 0.61 PD2: 67.19 ± 0.75 NDC2: 67.44 ± 1.12 HC2: 66.87 ± 0.91	Plasma	Plasma miRNA and PBL RNA extraction qRT-PCR Statistical analysis	miR-132 expression was significantly increased PD vs. healthy controls and controls with other neurological conditions. miR-132 expression was negatively correlated to the expression of Nurr1.	<0.05 *	Yes (validation cohort)
Chen et al., 2018 (China) [30]	Case–control PD patients: (*n* = 25) Males/Females: (*n* = 16/9) Controls: (*n* = 25) Males/Females: (*n* = 16/9) Ethnicity: N/A	PD: 64.96 ± 8.66 Controls: Age matched to be at ± 5 years of PD patients age	Plasma	RNA extraction Synthesis of cDNA miRNA expression Profiling analysis Data analysis	Eleven upregulated miRNAs in plasma: let-7g, miR-1, miR-10b, miR-144, miR-150, miR-29a, miR-34c, miR-382, miR-422a, miR-433, miR-539. Fourteen downregulated miRNAs in plasma: let-7a, let-7f, miR-125b, miR-130a, miR-130b, miR-142-3p, miR-185, miR-200a, miR-21, miR-222, miR-30a, miR-423-5p, miR-485-5p, miR-874.	All miRNAs listed <0.05 *	Yes (panel by other studies [39,70,71,72,73,74,75,76,77,78])
Khoo et al., 2012 (USA) [39]	Case–control Cohort 1: (*n* = 64) PD patients: (*n* = 32) Males/Females: (*n* = 16/16) Controls: (*n* = 32) Males/Females: (*n* = 15/17) Cohort 2: (*n* = 72) PD patients: (*n* = 42) Males/Females: (*n* = 20/22) Controls: (*n* = 30) Males/Females: (*n* = 10/20) Cohort 3: (*n* = 38) PD patients: (*n* = 30) Males/Females: (*n* = 16/14) Controls: (*n* = 8) Males/Females: (*n* = 3/5) Ethnicity: N/A	PD 1: 65 (66 ± 11)/69 (67 ± 11) Controls 1: 67 (65 ± 10)/68 (62 ± 17) PD 2: 69 (68 ± 6)/73 (72 ± 8) Controls 2: 65 (64 ± 15)/63 (59 ± 14) PD 3: 66 (68 ± 10)/73 (71 ± 7) Controls 3: 71 (71 ± 3)/73 (73 ± 4)	Plasma	RNA isolation miRNA expression microarrays Statistical analyses qRT-PCR Biomarkers evaluation	Five dysregulated miRNA pairs: miR-1826/miR-450b-3p, miR-506/miR-1253, miR-200a/miR-455-3p, miR-192/miR-485, miR-488/miR-518c. Three additional sole miRNA candidate biomarkers detected: miR-222, miR-505, miR-626.	0.0004 * 0.0001 * 0.0001 *	Yes (partially replicated by other study [30])
Ravanidis, Bougea, Papagiannakis, Koros, et al., 2020 (Greece) [19]	Case–control PD patients: (*n* = 109) Males/Females: (*n* = 57/52) Controls: (*n* = 92) Males/Females: (*n* = 33/59) Ethnicity: N/A	iPD: 64.22 ± 10.41 Controls: 57.10 ± 12.01	Plasma	miRNA isolation qRT-PCR Statistical analysis Pathway analysis	Twelve miRNAs tested and four found to be significantly altered: miR-22-3p, miR-139-5p, miR-154-5p, miR-330-5p.	0.007 * 0.021 * 0.038 * 0.028 *	Yes (validation of previous study by the same researchers [20])
Y. Chen et al., 2017 (China) [55]	Case–control PD patients: (*n* = 169) Males/Females: (*n* = 81/88) ET patients: (*n* = 60) Males/Females: (*n* = 32/28) Controls: (*n* = 170) Males/Females: (*n* = 83/87) Ethnicity: N/A	PD: 61.9 ± 5.1 ET: 61.5 ± 7.2 Controls: 61.6 ± 3.3	Plasma	MicroRNA microarray qRT-PCR CCK-8 Statistical analysis	Seven dysregulated miRNAs detected, with six of the being significant: miR-34c-3p, miR-148b-5p, let-7i-3p, miR-4639-5p, miR-34a-3p, miR-181a-5p, miR-30a-5p. miR-4639-5p found to regulate DJ-1 expression. Cells with overexpressed miR-4639-5p showed decreased viability.	<0.01 * <0.001 * <0.001 * <0.001 * 0.195 <0.001 * <0.001 *	Yes (cell model)
X. Zhang et al., 2017 (China) [57]	Case–control PD patients: (*n* = 46) Males/Females: (*n* = 22/24) Controls: (*n* = 49) Males/Females: (*n* = 22/27) Ethnicity: Chinese	PD: 63.13 ± 1.46 Controls: 60.35 ± 1.16	Plasma	RNA extraction qRT-PCR Pathway and gene ontology analyses of miRNA targets Supervised Learning Algorithms Statistical analysis	miR-433 and miR-133b were significantly downregulated in PD.	0.003 * 0.006 *	No
Nie et al., 2020 (China) [60]	Case–control PD patients: (*n* = 7) Males/Females: (*n* = 1/6) AD patients: (*n* = 5) Males/Females: (*n* = 1/4) Controls: (*n* = 20) Males/Females: (*n* = 10/10) Ethnicity: N/A	PD: 61.86 (47-74) AD: 67.8 (61–76) Controls: 34.45 (22–60)	Plasma	RNA extraction RNA sequencing Data analysis Target prediction KEGG pathway analysis Statistical analysis	Thirty-seven miRNAs dysregulated in AD: miR-197-3p, miR-576-5p, miR-1468-5p, miR-375, let-7e-5p, miR-483-3p, miR-3173-5p, miR-320e, miR-197-5p, miR-193b-5p, miR-6749-3p, miR-20a-5p, miR-191-3p, miR-4659a-3p, let-7b-3p, miR-17-5p, miR-3591-3p, miR-125a-5p, miR-204-5p, miR-122-5p, miR-19b-3p, miR-183-5p, let-7b-5p, miR-22-3p, miR-151a-5p, miR-27b-3p, miR-21-5p, miR-27a-3p, miR-146a-5p, miR-28-3p, miR-379-5p, miR-23a-3p, miR-199a-3p, miR-369-5p, miR-382-5p, miR-378i, miR-423-5p. Twenty dysregulated in PD samples: miR-197-3p, miR-576-5p, miR-1468-5p, miR-375, let-7e-5p, miR-211-5p, let-7e-3p, miR-122-3p, miR-941, miR-30d-5p, miR-192-5p, miR-93-5p, miR-425-5p, miR-99b-5p, let-7i-5p, miR-652-3p, miR-4732-3p, miR-6131, miR-3184-3p, miR-378g. Five were common: miR-197-3p, miR-576-5p, miR-1468-5p, miR-375, let-7e-5p.	<0.05 *	No
Li et al., 2024 (China) [62]	Case–control PD patients: (*n* = 53) Males/Females: (*n* = 25/28) iRBD patients (*n* = 56) Males/Females: (*n* = 34/22) Controls: (*n* = 60) Males/Females: (*n* = 35/25) Ethnicity: Chinese	PD: 63.0 ± 9.0 RBD: 64.0 ± 7.3 Controls: 63.5 ± 9.0	Plasma	Clinical assessment EV-RNA extraction EV isolation construction of cDNA TEM NTA Western Blot RNA sequencing Statistical analysis	In PD samples, a downregulation of 5 miRNAs: miR-96-5p, miR-155-5p, miR-150-5p, miR-150-3p, miR-3615. Upregulation of 10 miRNAs compared to controls: miR-27b-3p, miR-199a-5p, miR-151a-3p, miR-584-5p, miR-889-3p, miR-619-5p, miR-130b-5p, miR-197-3p, miR-4433b-5p, miR-4433a-3p.	NR	No
Wu et al., 2022 (China) [66]	Case–control PD patients: (*n* = 75) Males/Females: (*n* = 44/31) Controls: (*n* = 73) Males/Females: (*n* = 33/40) Ethnicity: N/A	PD: 68.0 (62.0–72.0) Controls: 67.0 (64.0–70.5)	Plasma	RNA extraction qRT-PCR Clinical evaluation Statistical analysis	miR-153 and miR-223 being notably reduced in PD plasma, while miR-7 was not significantly dysregulated.	0.006 * <0.001 * 0.546	Yes (results replicated by other included study [22])
Cressatti et al., 2020 (Canada) [22]	Case–control iPD patients: (*n* = 84) Males/Females: (*n* = 49/35) Controls: (*n* = 83) Males/Females: (*n* = 39/44) Ethnicity: N/A	iPD: 71.39 (1.38) Controls: 67.31 (1.04)	Saliva	Quantification of miRNA expression levels ELISA Statistical analysis	Significant downregulation of miR-153 and miR-223.	0.01 * 0.02 *	Yes (results replicated by other included study [66])
Jiang et al., 2021 (China) [49]	Case–control PD patients: (*n* = 50) Males/Females: (*n* = 19/31) MSA patients: (*n* = 20) Males/Females: (*n* = 6/14) ET patients: (*n* = 20) Males/Females: (*n* = 8/12) Controls: (*n* = 30) Males/Females: (*n* = 14/16) Ethnicity: N/A	PD: 63.62 ± 11.65 MSA: 63.00 ± 7.74 ET: 64.70 ± 9.07 Controls: 59.67 ± 11.18	Saliva	Microarray analysis Quantification of miRNA expression levels Statistical analysis	miR-29a-3p and miR-29c-3p were significantly reduced in expression, while miR-6756-5p was significantly upregulated.	0.004 * 0.027 * 0.032 *	Yes (partially validated by other included studies [24,45,65])
Chen et al., 2020 (China) [61]	Case–control PD patients: (*n* = 30) Males/Females: (*n* = 20/10) Controls: (*n* = 30) Males/Females: (*n* = 16/14) Ethnicity: N/A	PD: 63.20 ± 10.17 Controls: 59.57 ± 12.83	Saliva	RNA extraction qRT-PCR Statistical processing	miR-874 and miR-145-3p were detectable in most samples and found to regulate the expression of DJ-1.	NR	No
Ardashirova et al., 2022 (Russia) [65]	Case–control PD patients: (*n* = 70) Males/Females: (*n* = 35/35) Controls: (*n* = 40) Ethnicity: N/A	PD: 60.5 ± 11.8 Controls: NR	Leukocyte	RNA isolation qRT-PCR Statistical analysis	Five miRNAs significantly dysregulated: miR-7-1-5p, miR-29a-3p, miR-29c-3p, miR-30c-1-5p, miR-185-5p.	0.024 * 0.003 * 0.003 * 0.043 * 0.017 *	Yes (validated by other included studies [49,58])
Soreq et al., 2013 (Israel) [48]	Case–control PD patients: (*n* = 7) Males/Females: (*n* = 7/0) Controls: (*n* = 6) Males/Females: (*n* = 6/0) Ethnicity: N/A	NR	Leukocyte	RNA extraction RNA sequencing Mapping to miRBase and to human reference genome differential expression analysis Affymetrix HJAY Splice Junction Microarray HJAY Microarray Profiling, database Construction and analysis Brain transcriptome Microarray analysis Exon Microarrays Hybridization Cellular Lineage Analysis miRNA target predictions	Significant changes were found in the expression of 16 miRNAs pre-DBS treatment: miR-320a, miR-320b, miR-320c, miR-769, miR-92b, miR-16, miR-199b, miR-1274b, miR-21, miR-150, miR-671, miR-1249, miR-20a, miR-18b, miR-378c, miR-4293. Post treatment: miR-320a, miR-320b, miR-320c, miR-769, miR-92b, miR-16, miR-199b, miR-1274b, miR-21, miR-150, miR-671.	<0.05 *	Yes (pathway analysis)
Marques et al., 2017 (Netherlands) [26]	Case–control PD patients: (*n* = 28) Males/Females: (*n* = 21/7) MSA: (*n* = 17) Males/Female: (*n* = 13/4) Controls: (*n* = 28) Males/Female: (*n* = 15/13) Ethnicity: N/A	PD: 54.5 ± 10.4 MSA: 62.5 ± 9.7 Controls: 62.9 ± 8	CSF	RNA isolation qRT-PCR Data analysis	Ten miRNAs were screened. miR-205 and miR-24 could distinguish between controls and PD, while miR-24, miR-19a, miR-19b, and miR-34c could distinguish between MSA and controls.	<0.001 * <0.001 * <0.001 * <0.05 * <0.05 * <0.05 *	No
Qin et al., 2021 (China) [32]	Case–control PD patients: (*n* = 15) Males/Females: (*n* = 9/6) AD patients (*n* = 11) Males/Females: (*n* = 7/4) Controls: (*n* = 16) Males/Females: (*n* = 11/5) Ethnicity: N/A	PD: 70.6 ± 12.1 AD: 72.1 ± 10.8 Controls: 70.2 ± 15.8	CSF	RNA extraction qRT-PCR Statistical analysis	Concentration of miR-626 in the CSF was significantly decreased compared in both patients with AD and controls.	0.0018 * 0.0429 *	No
Gui et al., 2015 (China) [37]	Case–control Cohort 1: PD patients: (*n* = 47) Males/Females: (*n* = 25/22) AD patients: (*n* = 28) Males/Females: (*n* = 15/13) Controls: (*n* = 27) Males/Females: (*n* = 9/18) Cohort 2: PD patients: (*n* = 78) Males/Females: (*n* = 41/37) AD patients: (*n* = 53) Controls: (*n* = 35) Ethnicity: N/A	PD 1: 63 ± 9 (45–77) AD 1: 65 ± 12 (40–78) Controls 1: 60 ± 13 (42–79)	CSF	Exosome isolation Exosome characterization Electron Microscopy RNA processing miRNA profiling miRNA target prediction pathway analysis TaqMan miRNA Assay qRT-PCR Statistical analysis	Twenty-seven differentially expressed in CSF of patients with PD compared to controls: miR-1, miR-103a, miR-22, miR-29, miR-30b, miR-16-2, miR-26a, miR-331-5p, miR-153, miR-374, miR-132-5p, miR-119a, miR-485-5p, miR-127-3p, miR-126, miR-409-3p, miR-433, miR-370, let-7g-3p, miR-151, miR-28, miR-301a, miR-873-3p, miR-136-3p, miR-19b-3p, miR-10a-5p, miR-29c. Seven were significantly different compared to AD: miR-16-2, miR-331-5p, miR-132-5p, miR-485-5p, miR-151, miR-136-3p, miR-29c.	0.0078 * 0.0084 * 0.0090 * 0.0047 * 0.0044 * 0.0039 * 0.0058 * 0.0082 * 0.0057 * 0.0095 * 0.0023 * 0.0061 * 0.0025 * 0.0035 * 0.0038 * 0.0039 * 0.0043 * 0.0069 * 0.0068 * 0.0073 * 0.0035 * 0.0054 * 0.0052 * 0.0068 * 0.0109 * 0.0017 * 0.0013 *	Yes (validation cohort)
Tan et al., 2021 (China) [47]	Case–control PD patients: (*n* = 7) Males/Females: (*n* = 3/4) Controls: (*n* = 4) Males/Females: (*n* = 1/3) Ethnicity: N/A	PD: 53 ± 5 Controls: 46 ± 10	CSF	RNA isolation Cell culture Cell Treatment Cell Transfection qRT-PCR TUNEL Assay Western Blot Dual Luciferase Reporter Gene Assay Immunofluorescence Flow Cytometry Statistical analysis	Twenty-one differentially expressed miRNAs in patients with PD CSF samples: miR-486-5p, miR-122-5p, miR-451a, miR-423-5p, let-7b-5p, miR-151a-3p, miR-320a, miR-574-5p, miR-206, miR-204-5p, miR-1298-5p, miR-320b, miR-1246, miR-1307-3p, miR-128-3p, miR-409-3p, let-7a-5p, miR-144-3p, let-7d-3p, miR-4508, miR-155-5p. When tested on SH-SY5Y cells, miR-409-3p was the only one found to be significantly dysregulated.	0.000009 * 0.00002 * 0.00004 * 0.0001 * 0.0003 * 0.0003 * 0.002 * 0.003 * 0.003 * 0.004 * 0.005 * 0.007 * 0.008 * 0.01 * 0.01 * 0.02 * 0.02 * 0.03 * 0.03 * 0.03 * 0.04 *	Yes (cell model)
Dos Santos et al., 2018 (Belgium) [64]	Case–control PD patients: (*n* = 40) Males/Females: (*n* = 20/20) Controls: (*n* = 40) Males/Females: (*n* = 20/20) Ethnicity: N/A	PD: 61 ± 1 Controls: 64 ± 1	CSF	RNA extraction RNA sequencing Ligand Binding Assay Measurement Biomarker panel identification Gene target analysis Statistical analysis	One hundred twenty-one miRNAs expressed in the first 3 years of PD development. Five miRNAs as the most viable set of biomarkers: let-7f-5p, miR-125a-5p, miR-151a-3p, miR-27a-3p, miR-423-5p.	<0.05 *	No
Nair & Ge, 2016 (USA) [40]	Case–control PD patients: (*n* = 12) Males/Females: (*n* = 6/6) Controls: (*n* = 12) Males/Females: (*n* = 6/6) Ethnicity: N/A	PD: 75.6 ± 8.4 Controls: 74.1 ± 11.6	Post-mortem brain tissue	RNA isolation RNA expression analysis Gene expression analysis qRT-PCR Functional network analysis	Post-mortem PD patient tissues six significantly upregulated miRNAs: miR-3195, miR-204-5p, miR-485-3p, miR-221-3p, miR-95, miR-425-5p. Seven significantly downregulated miRNAs: miR-155-5p, miR-219-2-3p, miR-3200-3p, miR-423-5p, miR-4421, miR-421, miR-382-5p.	0.041 * 0.009 * 0.045 * 0.049 * 0.028 * 0.017 * 0.0163 * 0.0390 * 0.0003 * 0.0085 * 0.0120 * 0.0231 * 0.0465 *	No
Yu et al., 2024 (China) [41]	Case–control Cohort 1: (*n* = 718) PD patients: (*n* = 302) Males/Females: (*n* = 167/135) MSA patients: (*n* = 119) Males/Females: (*n* = 59/60) PSP patients: (*n* = 21) Males/Females: (*n* = 11/10) Controls: (*n* = 276) Males/Females: (*n* = 150/126) Cohort 2: (*n* = 425) PD patients: (*n* = 208) Males/Females: (*n* = 110/98) Controls: (*n* = 217) Males/Females: (*n* = 121/96) Cohort 3: (*n* = 60) iRBD patients: (*n* = 30) Males/Females: (*n* = 22/8) Controls: (*n* = 30) Males/Females: (*n* = 17/13) Cohort 4: (*n* = 88) PD patients: (*n* = 88) Males/Females: (*n* = 55/33) Ethnicity: N/A	PD 1: 66 (36–85) MSA 1: 62 (41–80) PSP 1: 65 (56–77) Controls 1: n (49–76) PD 2: 66 (34–87) Controls 2: 60 (51–89) iRBD 3: 65 (43–78) Controls 3: 59 (55–71) PD 4: 68 (36–87)	Post-mortem brain tissue	miR-44438–NCMB preparation TEM analysis Nanoscale Flow Cytometry Fluorescence Staining ZetaView NTA Analysis Statistical analysis	The concentration of miR-44438 in PD sample EVs was significantly increased compared to controls. miRNA concentration was also altered based on disease stage.	<0.001 *	Yes (validation cohorts)
Cho et al., 2013 (USA) [42]	Case–control PD patients: (*n* = 8) PDD patients: (*n* = 8) Controls: (*n* = 7) Ethnicity: N/A	PD/PDD: 80 ± 6.9 Controls: 85 ± 6.6	Post-mortem brain tissue	Western Blot qRT-PCR LCM RNA isolation Immunostaining Light Microscopy Cell cultures Constructs Transfection Luciferase Assays Image quantification Statistical analysis	PD patient post-mortem brain tissue showed an increased expression of LRRK2 proteins and a decreased expression of miR-205. Human cell model experiments showed that miR-205 expression is connected to downregulation of LRRK2.	0.0017 *	Yes (cell model)
Dobricic et al., 2022 (Germany) [52]	Case–control PD patients: (*n* = 214) AD patients: (*n* = 99) Controls: (*n* = 138) Ethnicity: N/A	NR	Post-mortem brain tissue	RNA extraction qPCR Statistical analysis	miR-132-3p was downregulated in PD and AD post-mortem brain tissue.	4.89 × 10^−6^ * 3.20 × 10^−24^ *	No
Hoss et al., 2016 (USA) [56]	Case–control/cohort PD patients: (*n* = 18) Males/Females: (*n* = 18/0) PDD patients: (*n* = 11) Males/Females: (*n* = 11/0) Controls: (*n* = 33) Males/Females: (*n* = 33/0) Ethnicity: N/A	PD: 76.1 ± 8.9 PDD: 79.9 ± 9.0 Controls: 68.1 ± 14.8	Post-mortem brain tissue	Small RNA sequencing Statistical analysis	Sixty-four miRNAs downregulated, while sixty-one were upregulated in PD brain tissue. One hundred-five were significant. No significant changes between standard PD and PDD patients.	<0.05 *	No
Margis et al., 2011 (Brazil) [46]	Case–control/cohort Drug-naïve PD patients: (*n* = 8) Males/Females: (*n* = 4/4) EOPD: (*n* = 7) Males/Females: (*n* = 4/3) Controls: (*n* = 8) Males/Females: (*n* = 4/4) Ethnicity: N/A	Untreated PD: 66 (6.7) EOPD: 45 (8.7) Controls: 67 (8.0)	Blood	qPCR Data analysis	Untreated patients with PD had a significantly lower expression of these miRNAs compared to the other groups: miR-1, miR-22, miR-29a.	<0.05 * 0.07 * <0.05 *	No
Tolosa et al., 2018 (Spain) [43]	Case–control/cohort PD patients: (*n* = 3) Males/Females: (*n* = 0/3) L2PD patients: (*n* = 3) Males/Females: (*n* = 1/2) Controls: (*n* = 4) Males/Females: (*n* = 2/2) Ethnicity: N/A	NR	iPSCs	Generation of iPSCs miRNA isolation miRNA expression analysis Identification of differentially expressed miRNAs qRT-PCR Enrichment analysis association of miRNA and gene expression Functional network analysis	Five miRNAs upregulated in PD patient-derived iPSCs: miR-9-5p, miR-135a-5p, miR-135b-5p, miR-449a, miR-449b-5p Five miRNAs downregulated in PD patient-derived iPSCs: miR-141-3p, miR-199a-5p, miR-299-5p, miR-518e-3p, miR-519a-3p.	NR	Yes (enrichment and functional network analysis)
Scheper et al., 2023 (Netherlands) [21]	Case–control PD4 patients: (*n* = 16) Males/Females: (*n* = 8/8) PD 5/6 patients: (*n* = 9) Males/Females: (*n* = 6/3) PDD 5/6 patients: (*n* = 19) Males/Females: (*n* = 13/6) Controls: (*n* = 19) Males/Females: (*n* = 9/10) Ethnicity: N/A	PD4: 71 ± 11.75 PD 5/6: 73.3 ± 9.15 PDD 5/6: 78.8 ± 6.01 Controls: 89.43 ± 11.22	Post-mortem brain tissue, CSF	RNA sequencing Read quality and alignment miRNA target prediction RNA isolation qRT-PCR Immunohistochemistry Cell cultures and transfection Western Blot Statistical analysis	miRNAs significantly dysregulated: let-7e-3p, miR-424-3p, miR-543.	< 0.05 * < 0.05 * < 0.05 *	Yes (cell model)
Braunger et al., 2024 (Germany) [67]	Cohort Sporadic PD: (*n* = 10) LRRK2 PD: (*n* = 6) LRRK2 carriers: (*n* = 4) Controls: (*n* = 11) Ethnicity: N/A	Sporadic PD: 64.5 ± 10.9 LRRK2 PD: 66.8 ± 8.7 LRRK2 carriers: 52.3 ± 19.6 Controls: 60.1 ± 14.5	Plasma, CSF	RNA isolation qRT-PCR Processing of raw data and visualization Integration of CSF and plasma data sets Identification of discriminatory miRNAs Target prediction enrichment analysis	miR-29c-3p, miR-128-3p, miR-424-5p, miR-223-3p were found to overlap between LRRK2 PD, LRRK2 carriers and sporadic PD. miRNAs could also discriminate between these models.	NR	Yes (panel by other studies [11,15,17,26,37,39,40,54,55,64,79,80,81,82,83,84,85,86,87,88,89,90,91,92,93,94])
Starhof et al., 2019 (Denmark) [44]	Case–control Cohort 1: (*n* = 40) PD patients: (*n* = 10) MSA patients: (*n* = 10) PSP patients: (*n* = 10) Controls: (*n* = 10) Cohort 2 (*n* = 121) PD patients: (*n* = 37) Males/Females: (*n* = 25/12) MSA patients: (*n* = 29) Males/Females: (*n* = 10/19) PSP patients: (*n* = 32) Males/Females: (*n* = 22/10) Controls: (*n* = 23) Males/Females: (*n* = 11/12) Ethnicity: N/A	PD 2: 66.3 (12.0) MSA 2: 63.2 (11.9) PSP: 69.4 (5.6) Controls 2: 41.5 (17.6)	Plasma, CSF	RNA isolation miRNA analysis CSF/α-synuclein Quantitation Statistical analysis	Eight miRNAs were differentially expressed at significant levels in the CSF of patients: let-7b-5p, miR-106b-5p, miR-184, miR-218-5p, miR-331-5p, miR-34c-3p, miR-7-5p, miR-99a-5p.	<0.001 * 0.003 * 0.007 * 0.007 * 0.030 * 0.032 * 0.047 * 0.047 *	Yes (validation cohort)

Abbreviations: Advanced Parkinson’s Disease: aPD, Alzheimer’s Disease: AD, Brain-Derived Neurotrophic Factor: BDNF, cerebrospinal fluid: CSF, De Novo Parkinson’s Disease: dnPD, Early Onset Parkinson’s Disease: EOPD, essential tremor: ET, Fibronectin type III domain-containing protein 5: FNDC5, Glucosylceramidase Beta: GBA, Idiopathic Parkinson’s Disease: iPD, Induced Pluripotent Stem Cells: iPSCs, isolated rapid eye movement sleep behavior disorder: iRBD, Leucine–Rich Repeat Kinase 2: LRRK2, mild cognitive impairment: MCI, multiple system atrophy: MSA, neurological disease: ND, nuclear receptor-related 1: Nurr1, peripheral blood mononuclear cells: PBMCs, Parkinson’s Disease: PD, Parkinson’s Disease Dementia: PDD, Peroxisome Proliferator-Activated Receptor Gamma Coactivator 1-Alpha: PGC1a, Prodromal Parkinson’s Disease: pPD, Progressive Supranuclear Palsy: PSP. * Indicates statistical significance within the studies included.

## Data Availability

No new data were created or analyzed in this study. Data sharing is not applicable to this article.

## Supplementary Materials

The following supporting information can be downloaded at: https://www.mdpi.com/article/10.3390/ijms262412164/s1.

## Abbreviations

PD	Parkinson’s Disease
iPD	Idiopathic Parkinson’s Disease
fPD	Familial Parkinson’s Disease
SNCA	Alpha-Synuclein
VPS35	Vesicle Protein Sorting 35
PINK1	PTEN Induced Kinase 1
PRKN	Parkin
PARK7	Parkinsonism Associated Deglycase
PLA2G6	Phospholipase A2 Group VI
GBA	Glucosylceramidase Beta
LRRK2	Leucine–Rich Repeat Kinase 2
miRNA	Micro-RNA
AD	Alzheimer’s Disease
ALS	Amyotrophic Lateral Sclerosis
MS	Multiple Sclerosis
BBB	Blood–Brain Barrier
iRBD	Isolated Rapid Eye Movement Sleep Behavior Disorder
CSF	Cerebrospinal Fluid
PBMCs	Peripheral Blood Mononuclear Cells
iPSCs	Induced Pluripotent Stem Cells
AKT3	AKT serine/threonine kinase 3
PGC1α	Peroxisome proliferator-activated-receptor-γ cofactor 1-alpha
MSA	Multiple System Atrophy
ET	Essential Tremor
MEST	Mesoderm-specific transcript
SPARC	Secreted protein acidic and rich in cysteine
BACE1	Beta-site Amyloid precursor protein Cleaving Enzyme 1
KLF7	Kruppel-like factor 7
PTEN	Phosphatase and TENsin homolog
IL-8	Interleukin-8
DOAJs	Directory of Open Access Journals
SSRN	Social Science Research Network
